# The Nervous System Development Regulator Neuropilin-1 as a Potential Prognostic Marker and Therapeutic Target in Brain Cancer

**DOI:** 10.3390/cancers15204922

**Published:** 2023-10-10

**Authors:** Eduardo Mello Rodrigues, Allan Fernando Giovanini, Carmen Australia Paredes Marcondes Ribas, Osvaldo Malafaia, Rafael Roesler, Gustavo R. Isolan

**Affiliations:** 1 Graduate Program in Principles of Surgery, Mackenzie Evangelical University, Curitiba 80730-000, PR, Brazil; eduardor@feevale.br (E.M.R.);; 2 The Center for Advanced Neurology and Neurosurgery (CEANNE), Porto Alegre 90560-010, RS, Brazil; 3 Department of Pharmacology, Institute for Basic Health Sciences, Federal University of Rio Grande do Sul, Porto Alegre 90035-003, RS, Brazil; 4 Cancer and Neurobiology Laboratory, Experimental Research Center, Clinical Hospital (CPE-HCPA), Federal University of Rio Grande do Sul, Porto Alegre 90035-003, RS, Brazil; 5 National Science and Technology Institute for Children’s Cancer Biology and Pediatric Oncology—INCT BioOncoPed, Porto Alegre 90035-003, RS, Brazil; 6 Spalt Therapeutics, Porto Alegre 90560-010, RS, Brazil

**Keywords:** neuropilin-1, NRP1, medulloblastoma, glioblastoma, glioma, brain tumor

## Abstract

**Simple Summary:**

Dysregulation of molecular mechanisms that regulate nervous system development may be at the origin of brain tumors. We reviewed the role of neuropilin-1 (NRP1), a transmembrane glycoprotein that directs neuronal migration during development, in brain cancer types that afflict children or adults. We also present gene expression analysis showing that higher NRP1 expression is associated with higher grade tumors compared to lower grade tumors or non-tumoral brain tissue. Importantly, higher tumoral NRP1 mRNA content in glioblastoma (GBM) is associated with a worst patient prognosis. This evidence supports the view that NRP1 is a potential prognostic biomarker and therapeutic target in brain tumors.

**Abstract:**

Neuropilins are transmembrane glycoproteins that regulate developmental processes in the nervous system and other tissues. Overexpression of neuropilin-1 (NRP1) occurs in many solid tumor types and, in several instances, may predict patient outcome in terms of overall survival. Experimental inhibition of NRP1 activity can display antitumor effects in different cancer models. Here, we review NRP1 expression and function in adult and pediatric brain cancers, particularly glioblastomas (GBMs) and medulloblastomas, and present analyses of NRP1 transcript levels and their association with patient survival in GBMs. The case of NRP1 highlights the potential of regulators of neurodevelopment as biomarkers and therapeutic targets in brain cancer.

## 1. Introduction

Neuropilins were first discovered as proteins influencing nervous system development and later emerged as regulators of the development and function of other tissues, including the cardiovascular and immune systems. A role for neuropilins in cancer biology has also been described through influences in angiogenesis and vascular and lymphatic biology as well as by directly modulating signaling within cancer cells. Increasing evidence indicates that neuropilins, particularly neuropilin-1 (NRP1), can affect the progression of different types of brain tumors, and modification of NRP1 activity has been investigated as an experimental therapeutic approach. In this article, we review the current knowledge on the expression and role of NRP1 in brain cancers and its potential as a target in experimental neurooncology.

## 2. Neuropilin-1

### 2.1. Structure

Neuropilins are transmembrane glycoproteins structurally organized in three domains that act as non-tyrosine kinase surface proteins. They occur specifically in vertebrates in two homologous isoforms, NRP1 and neuropilin-2 (NRP2), which share 44% sequence homology with each other [1,2,3,4]. The three protein domains that constitute neuropilins are the N-terminal extracellular, transmembrane, and intracellular domains. The extracellular domain contains three subdomains, a1a2 (CUB), b1b2, and c. Extracellular domains participate in ligand binding, whereas domain c mediates homo- and hetero-dimerization [4,5]. NRP1 in humans measures 130–140 kDa and is encoded by the *NRP1* gene, which is located on chromosome 10p11.22. The *NRP1* gene spans over 120 kb and contains 17 exons. Alternative transcript splicing gives rise to additional protein isoform variants [2,3,4,5,6].

### 2.2. Ligands and Co-Receptors

NRP1 functions as a co-receptor capable of binding several different ligands to ultimately influence multiple signaling pathways. NRP1 is devoid of kinase activity by itself but can stimulate the kinase function of its co-receptors. The first receptors identified for NRP1 were semaphorins (SEMAs), particularly those belonging to SEMA class 3 (SEMA 3), which are further classified into seven soluble proteins, SEMA3A–SEMA3G. Each SEMA 3 member shows different affinities for NRP1 [5]. NRP1 binds to SEMA through its a1, a2, and b1 domains and enhances the binding of SEMA 3 to plexins, giving rise to a complex formed by NRP1, SEMAs, and the plexins and influencing embryonic development, immune responses, and axon guidance [4,7,8,9]. NRP1 also acts as a receptor for several families of growth factors and their receptors, notably those of the vascular endothelial growth factor (VEGF) family, which consists of VEGF A, B, C, and D, in addition to placental growth factor (PlGF). NRP1 binds different isoforms of VEGF-A through its b1b2 extracellular domain and is also a co-receptor for at least two VEGF receptor types, VEGFR-1 and VEGFR-2, thus regulating angiogenesis [10,11]. Platelet-derived growth factor (PDGF)-D binds directly to NRP1 and induces translocation of NRP1 to cell–cell junctions in endothelial cells, affecting how NRP1 affects signaling mediated by VEGF-A/VEGFR-2 [12]. NRP1 associates with phosphorylated PDGF receptors (PDGFR) to influence cell proliferation and migration [13]. Fibroblast growth factor (FGF), its receptor FGFR-1, hepatocyte growth factor (HGF), and the transforming growth factor β (TGF-β) are all additional targets of NRP1. The intracellular domain of NRP1 interacts with cytoplasmic proteins, including synectin [4,14]. These multiple actions enable NRP1 as a regulator of cell signaling and tissue development and function, notably in the vascular, immune, and nervous systems [15,16,17,18,19].

## 3. NRP1 Regulation of Nervous System Development, Damage, and Reparation

Several lines of evidence demonstrate a role for NRP1 in regulating the normal development of the peripheral and central nervous system as well as other tissues. NRP1 plays a crucial role in directing neural crest cells that are precursors of peripheral neurons during development [20]. Chimeric mouse embryos overexpressing NRP1 obtained from embryonic stem cell clones that constitutively express exogenous neuropilins show a range of morphological anomalies, including dilated blood vessels, abnormal hearts, ectopic sprouting, and defasciculation of nerve fibers [21]. NRP1 mutant mouse embryos show vascular defects that include neural and vascularization impairment [22]. Expression of NRP1 in the rat olfactory bulb is significantly higher during early development compared to adulthood [23]. In brain stem neurons, NRP1 is a target gene of the orphan nuclear receptor Nurr1 in regulating neuronal development [24].

During neurodevelopment, precise connections between neurons and their appropriate targets are established. The leading edge of an axon, called the growth cone, drives the axon to its targets. This guidance process is mediated by several attractive and repulsive biochemical cues [25]. Through its action on SEMA 3, NRP1 stimulates neuronal growth cone collapse, chemorepulsion of neurites, and guidance of sensory afferent innervation [7]. Experiments using SEMA 3E null mutant mice to examine the role of SEMA 3E/plexinD1 in the development of descending axon tracts in the brain show that, in corticofugal and striatonigral neurons lacking NRP1, SEMA 3E acts as a repellent, whereas in NRP1-expressing subiculo-mammillary neurons, it acts as an attractant. This conversion of repulsive signaling to attraction during brain development is mediated by the extracellular domain of NRP1 [26].

Further studies show that neuropilins direct distinct neuronal populations to different brain areas. Migrating cortical interneurons expressing neuropilins and their receptors, including SEMA 3A and SEMA 3F, are directed to the cortex, whereas neurons lacking neuropilins go to the striatum. SEMA 3A and SEMA 3F thus mediate a chemorepulsive signal from the striatum, which is enabled by neuropilins in migrating cortical neurons, establishing a migratory route to the cortex [27]. This role of neuropilins depends on their interaction with the Robo1 protein [28]. It was later demonstrated that NRP1 regulates not only migration but also the generation of cortical interneurons in the ventral telencephalon by interacting with SEMA 3A [29].

In the adult nervous system, NRP1/SEMA 3 may play a role in the regeneration and plasticity of sensory neurons [30]. NRP1 is a direct target of the nuclear transcription factor E2F1 in regulating axonal damage and neuronal death associated with cerebral ischemia [31]. In in vitro and in vivo rat models of cerebral ischemia, a sharp increase in NRP1 expression is observed in the neural tissue. Expression of adeno-associated viral (AAV)-NRP1 markedly ameliorates ischemia-induced impairment of motor function, rescues changes in mitochondrial morphology, and improves mitochondrial oxidative stress and bioenergetic deficits [32]. Expression of NRP1 in the spinal cord is increased in a mouse model of amyotrophic lateral sclerosis [33]. In an experimental spinal cord injury, NRP1 mediates the pruning of corticospinal tract fibers allowing motor recovery [34].

## 4. NRP1 Regulation of Non-Tumoral Stem Cell Function

An aspect of NRP1’s function related to its regulation of nervous system development and plasticity is its ability to influence the proliferation and differentiation of stem cells. Embryonic stem cells expressing NRP1 can give rise to precursors of neural cells as well as precursors of endothelial cells [35]. NRP1 expression allows the identification of endothelial precursors in murine and human embryonic stem cells prior to expression of CD31 or CD34, and blocking the VEGF and SEMA binding functions of NRP1 impairs the differentiation of precursors to endothelial cells [36]. NRP1 also promotes the arterial differentiation of murine pluripotent stem cells into vascular precursors [37]. In human mesenchymal stem cells, NRP1 regulates signaling, proliferation, migration, and network assembly by associating with phosphorylated PDGFR [13]. Human mesenchymal stromal cells modified with NRP1 stimulate the expansion of hematopoietic stem and progenitor cells with enhanced functionality, indicating a regulatory role for NRP1 in hematopoiesis [38]. Human adipose-derived stem cells display an increased expression of keratinocyte growth factor (KGF) during adipogenic differentiation, and NRP1 mediates KGF activation in these cells [39]. In yet another example of stem cell regulation by NRP1, it modulates odontoblast differentiation of dental pulp stem cells by interacting with the FYN protein tyrosine kinase [40]. Together, these studies provide examples of NRP1 involvement in stem cell function and differentiation in different tissue types and through a range of cellular mechanisms.

## 5. NRP1 in Cancer: Peripheral Solid Tumors

### 5.1. NRP1 in Cancer Cell Function and Tumor Growth

Gene expression of NRP1 is often but not always higher in tumor cells compared to corresponding non-tumoral tissue [41,42]. Earlier experiments exploring how NRP1 influences cancer growth show that a soluble NRP1 (sNRP1) isoform used as a tool to inhibit NRP1 activity induces vascular damage, hemorrhage, and apoptosis in tumors from rat prostate cancer cells [43]. In the human pancreatic cell line FG, NRP1 overexpression enhances anoikis resistance and increases cell survival upon exposure to clinically relevant doses of cytotoxic chemotherapeutics gemcitabine and 5-Fluorouracil (5-FU). Conversely, NRP1 downregulation in Panc-1 cells markedly increases chemosensitivity. Moreover, overexpression of NRP1 increases constitutive mitogen-activated protein kinase (MAPK) activation [44].

Measuring NRP1 expression by immunohistochemistry and in situ hybridization shows ubiquitous expression in colon adenocarcinoma tumors, and reverse transcriptase-polymerase chain reaction analysis (RT-PCR) shows NRP1 mRNA expression in colon adenocarcinoma cell lines. Overexpression of NRP1 leads to increased tumor growth and angiogenesis in human colon cancer cells xenografted into nude mice. Moreover, conditioned medium from NRP1-transfected colon cancer cells enhances endothelial cell migration. Regulation of NRP1 levels in colon cancer cells may involve epidermal growth factor (EGF) and MAPK signaling [45]. Inhibition of NRP1 expression in WiD human colon adenocarcinoma cells induced by RNA interference decreases cell migration [46].

Expression of NRP1 is found in hepatocellular carcinoma (HCC) but not in normal hepatocytes. Blockade of NRP1 function induced by peptide N results in inhibition of vascular remodeling and tumor growth in mice with HCC [47]. Also, in experimental HCC, NRP1 targeting with microRNA-148b (miRNA-148b) reduces tumorigenicity [48]. Silencing NRP1 expression can inhibit the activation of hepatic stellate cells, which stimulate the proliferation, migration, and invasion of primary liver cancer cells [49]. As shown with the gene network analysis of epithelial ovarian cancer, NRP1 is also targeted by other miRNAs, including miRNA-130a and miRNA-130b [50].

Systemic administration of an antibody that blocks VEGF binding to the b domain of NRP1 (anti-NRP1B) slows tumor growth in NRP1-expressing lung carcinoma [35]. Monoclonal antibodies against NRP1 have an additive effect with anti-VEGF therapy to inhibit the growth of non-small cell lung cancer (NSCLC) xenografts in mice [51]. Lung cancer cells expressing NRP1 show increased levels of stemness markers and tumor-initiating properties, suggesting that NRP1 may be a feature of cancer stem cells (CSCs) [52]. Consistent with this possibility, a subpopulation of breast cancer cells expressing CSC markers also express NRP1, and an anti-NRP1 antibody inhibits the formation of breast CSC-enriched tumorspheres. NRP1-triggered stimulation of breast tumorsphere formation is dependent on nuclear factor kappa B (NF-κB) [53]. Combined knockdown of NRP-1 and VEGF inhibits the proliferation, migration, and invasion while enhancing the apoptosis of MDA-MB-231 human breast cancer cells. In contrast, induction of NRP1 over expression promotes the proliferation, migration, and invasion of MCF-7 breast cancer cells [54]. NRP1 protein concentrations are significantly higher in breast cancer patients with lymph node involvement compared to those without lymph node involvement [55]. Overexpression of NRP1 in BT-747 breast cancer cells leads to upregulation of the oncogene Tenascin-C and is accompanied by an increase in cellular tumorigenic behavior and downregulated breast cancer resistance protein (BCRP/ABCG2), resulting in enhanced sensitivity to chemotherapy [56]. NRP1 expression is increased by glycoprotein NMB (GPNMB), and NRP1 and GPNMB cooperate to stimulate mammary tumor growth. GPNMB drives an increase in NRP1 levels, which in turn potentiates VEGF signaling in breast cancer cells to mediate the growth but not metastasis of these cells in an in vivo experimental model [57].

In melanoma cells, NRP1 expression promotes invasiveness partially through VEGFR-2. NRP1 expression is also sufficient to promote melanoma cell invasion accompanied by MMP-2 secretion. The ability of NRP1 to promote melanoma invasiveness involves Akt activation through its phosphorylation on T308 [58]. NRP1 also contributes to invasiveness in squamous cell carcinoma (SCC) of the esophagus. Interestingly, a higher molecular weight-modified NRP1 (mNRP1) type was identified in a large proportion of oesophageal SCC tumors, and its expression is associated with less lymph node metastasis and a better prognostic tumor–node–metastasis stage compared to mNRP1-negative tumors [59]. NRP1 depletion inhibits gastric cancer cell proliferation by inducing cell cycle arrest and upregulating p27 while downregulating cyclin E and cyclin-dependent kinase 2 in addition to suppressing cell migration by inhibiting focal adhesion kinase phosphorylation. Moreover, NRP1 inhibition hinders gastric tumor growth and lung metastasis by reducing cell proliferation and tumor angiogenesis. Furthermore, NRP1 knockdown by a shRNA lentivirus inhibits the growth of BGC823 gastric tumors [60]. Migration and invasion of human gastric cancer cells can be suppressed by an anti-neuropilin-1 monoclonal antibody, anti-NRP1 mAb, through Akt dephosphorylation [61].

A study using tumor biopsies from children with neuroblastomas (NBs) to assess the mRNA expression of neuropilin with quantitative RT-PCR showed that NRP1 and NRP2 mRNA and protein levels are higher in stages I-IV NB compared to non-tumor controls [62]. Knockdown of NRP1 promotes the migration and invasion of human neuroblastoma SK-N-AS cells through a mechanism involving stimulation of β1 integrin expression [63]. MG-63 osteosarcoma cells transfected to promote higher NRP1 expression display increased invasion capacity and cell survival after exposure to doxorubicin, whereas NRP1 downregulation in SaOS-2 cells increases chemosensitivity to doxorubicin [64]. Combined targeting of mammalian target of rapamycin (mTOR) signaling and VEGF/NRP1 using a liposomal formulation decorated with a proprietary tumor-targeting peptide that simultaneously delivers the mTOR inhibitor everolimus and the NRP1 inhibitor EG00229 reduces the growth of experimental clear cell renal cell carcinoma and related lung metastases [65]. NRP1 forms a complex with α6/β4-integrin and GIPC1 to activate FAK/Src signaling and promote stabilization of YAP1/∆Np63α to enhance the survival, invasion, and angiogenesis in epidermal cancer cells [66].

### 5.2. NRP1 and the Tumor Microenvironment

In addition to directly regulating cancer cell function and angiogenesis, NRP1 can interact with different components of the tumor microenvironment. For example, NRP1 on stromal myofibroblasts acts on soluble fibronectin to promote fibronectin fibril assembly and matrix stiffness, resulting in the stimulation of tumor growth. An increase in fibronectin fibril assembly depends on α5β1 integrin, glycosylation of serine 612 of the extracellular domain of NRP1, an intact intracellular NRP1 SEA domain, and associations between NRP1, GIPC, and the nonreceptor tyrosine kinase c-Abl [67]. In cervical cancer, NRP1 is critical for the activation of tumor-associated macrophages by a hypoxic tumor microenvironment [68]. SEMA 3A leads to stimulation of an NRP1/plexin A4 heterodimer to form an immunoregulatory receptor complex, resulting in an enhanced number and function of regulatory CD4+ T cells [69]. Regulatory T cells (Tregs) in the tumor microenvironment participate in the immunoregulation of cancer growth, producing immunosuppressive cytokines to support tumor growth, including interleukin-10 (IL-10). Tumor progression is accompanied by a reduction in Tregs that produce NRP1 [70], and NRP1 can partially mediate a reduction in Treg number and function induced by anti-angiogenic drugs [71]. NRP1 is upregulated in T regulatory cells from patients with pancreatic adenocarcinoma and colorectal cancer (CRC) metastasis to the liver. Thus, expression of NRP1 is significantly higher in CD3-positive and CD4-positive tumor-infiltrating lymphocytes (TILs) compared to peripheral blood mononuclear cells (PBMCs) in patients with CRC liver metastasis. In addition, NRP1 expression is induced in PBMCs co-cultured in vitro with tumor tissue [72,73]. Remarkably, in head and neck cancer, the prevalence of NRP1-positive Tregs in tumors is associated with poorer patient outcomes shown by reduced progression-free survival [74].

### 5.3. NRP1 Regulation of Resistance to Anticancer Therapies

NRP1 can modulate tumor resistance to different modalities of therapy. The NRP1 inhibitor EG00229 sensitizes A549 lung carcinoma cells to the cytotoxic chemotherapeutics paclitaxel and 5-FU [75]. Another inhibitor, EG3287, which displaces the binding of VEGF to NRP1, potentiates the effects of 5-FU, paclitaxel, and cisplatin on A549 and DU145 cells [76]. NRP1 can also contribute to the resistance to radiotherapy as demonstrated by experiments in non-small-cell lung cancer cells [77]. Radiation-resistant cellular models of lung adenocarcinoma show increased NRP1 expression, and the NRP1 inhibitor EG00229 reduces the expression levels and binding capacity of NRP1 [78]. NRP1 regulation of radioresistance in lung cancer involves downstream homeobox genes HOXA6 and HOXA9 [79], and microRNA-9 is able to restore radio-sensitivity in radioresistant lung cancer cells by targeting NRP1 [80]. NRP1 can also promote resistance to molecularly targeted therapies in cancer. Adaptative NRP1 expression is observed in melanoma cells after treatment with BRAF inhibitors and in breast cancer cells treated with HER2 inhibitors, resulting in an acquired resistance to therapy. In addition, NRP1 levels predict the efficacy of MET oncogene inhibitors in stomach and lung carcinoma cells. NRP1-mediated drug resistance involves the enhancement of JNK-dependent signaling leading to the upregulation of either the EGF receptor (EGFR) or insulin growth factor receptor (IGF1R), which in turn sustain the acquired resistance to BRAF, HER2, and MET inhibitors. NRP1-interfering molecules can improve the efficacy of targeted drugs and reverse the onset of resistance [81].

### 5.4. NRP1 as a Predictor of Prognosis

NRP1 expression may be a marker of poor prognosis in different cancer types [9]. For example, a high expression of NRP1 is significantly correlated with angiogenesis, an advanced tumor–node–metastasis stage, p T stage, node invasion, and poor postoperative overall survival in patients with pancreatic adenocarcinoma [82]. In patients with advanced colorectal carcinoma, those with tumors with high NRP1 immunohistochemical staining show a higher incidence of lymph node and liver metastasis, greater microvessel density, a higher number of proliferating cells, and fewer apoptotic cells compared to patients bearing tumors with low levels of NRP1. In addition, high NRP1 staining is associated with shorter patient survival [46]. Patients with NSCLC coexpressing both NRP1 and NRP2 show a poorer prognosis in comparison with patients with tumors lacking coexpression of both neuropilins [83]. Also, in patients with NSCLC, another study showed that patients with a high expression of NRP1 have shorter disease-free and overall survival [84]. In epithelial ovarian carcinoma, NRP1 levels are higher in tumors at an advanced stage and present lymph node metastasis and distant metastasis, and higher NRP1 expression strongly predicts shorter survival [85]. Upregulation of NRP1 is prognostic of metastatic progression and patient mortality in prostate cancer [86]. NRP1 is also a predictor of poorer prognosis in patients with oral SCC [87].

## 6. NRP1 in Brain Cancer

### 6.1. Glioblastoma

A glioblastoma (GBM) is the most prevalent and lethal type of primary brain tumor, accounting for half of newly diagnosed gliomas. Gliomas are a group of cancer types that together comprise about 80 percent of central nervous system (CNS) tumors in adults. [88]. The prognosis for GBM patients remains dismal, with a median overall survival of 12–15 months in spite of multimodal treatment combining surgical resection, radiotherapy, and chemotherapy with temozolomide [89,90].

Human malignant glioma cell lines express NRP1 mRNA and protein, as well as SEMA3A and SEMA3C [91]. NRP1 promotes experimental GBM progression [92,93]. U87MG GBM tumor xenografts overexpressing NRP1 show increased tumor growth and angiogenesis. NRP1-overexpressing U87MG cells show enhanced survival mediated by increased autocrine hepatocyte growth factor/scatter factor (HGF/SF)/c-Met signaling. NRP1 potentiates the activity of endogenous HGF/SF on cell survival and HGF/SF-promoted cell proliferation, whereas the inhibition of HGF/SF, c-Met, and NRP1 hinders NRP1-potentiated autocrine HGF/SF stimulation [92]. NRP1 is more highly expressed in human GBM and C6 rat glioma cells than in non-tumoral human brain tissue and primary rat astrocytes. RNAi-mediated knockdown of NRP1 reduces the proliferation of C6 glioma cells stimulated by the NRP1 ligand glia cell line-derived neurotrophic factor (GDNF) [94]. A synthetic peptide that acts as an antagonist of the NRP1 transmembrane domain (pTM-NRP1) reduces the growth of C6 GBM tumors in orthotopic and heterothopic in vivo models in rats and nude mice by inhibiting GBM cell proliferation and migration in addition to angiogenesis [95]. Overexpression of miR-124-3p inhibits GBM cell proliferation, migration, and tumor angiogenesis, resulting in GBM apoptosis and cell cycle arrest through NRP1 mediated activation of the phosphoinositide 3 kinase (PI3K)/Akt/NFκB pathway. In addition, anti-NRP1 mAb displays synergistic inhibitory effects with miR-124-3p overexpression in GBMs. Thus, miR-124-3p is an upstream inhibitor of NRP-1 in GBMs [96]. GBM cells responsive to VEGF-A signaling show a physical interaction between wild-type NRP1 and FMS-related receptor tyrosine kinase 1 (Flt-1), whereas VEGF-A-resistant GBMs show modified chondroitin-sulfated NRP-1 with no interaction with Flt-1, and eliminating the chondroitin sulfate modification in NRP-1 leads to re-sensitization to VEGF-A [97].

Patient-derived GBM cells expressing shRNAs for either VEGF or NRP1 show reduced stemness markers and neurosphere-forming capacity, and knockdown of VEGF and NRP1 inhibits the growth of patient-derived GBM xenografts in both zebrafish and mice and prolongs mouse survival. Moreover, NRP1 enhances GBM cell sensitivity to temozolomide [98]. An immunostaining study found that GBM CD133-expressing brain tumor stem cells (BTSCs) highly express NRP1, whereas differentiated GBM cells do not. Knockdown of NRP1 with shRNA suppresses SEMA3A-mediated inhibition of cell proliferation and increases invasion [99]. The viability, self-renewal capacity, and tumorigenicity of CD133+ BTSC GBM cells involve signaling through the VEGF-VEGFR2-NRP1 axis, which may counteract the effect of anti-angiogenic therapy with bevacizumab. Moreover, inhibition of NRP1 by shRNA-mediated knockdown reduces BTSC viability [100]. Both RNA interference-mediated silencing and CRISPR-mediated gene editing deletion of NRP1 strongly impair the invasive capacity of properties of patient-derived GBM BTSCs and their close localization to brain blood vessels without affecting BTSC expansion and self-renewal. These actions of NRP1 in BTSCs may depend on the expression of the β3 subunit integrin cell–extracellular matrix adhesive receptor [101]

RT-PCR of 37 glioma specimens, out of which 17 were grade IV GBM tumors, showed that NRP1 mRNA expression is higher in GBM than in non-tumoral tissue and lower-grade gliomas, and glioma patients with NRP1 overexpression (56.8%) showed a poorer prognosis indicated by reduced overall survival [102]. Moreover, NRP1 is an independent risk factor for both survival and recurrence in patients with GBMs, and high NRP1 mRNA expression is associated with shorter overall and disease-free survival, as shown by analysis of The Cancer Genome Atlas (TCGA) dataset [93]. NRP1 expression correlates with the mesenchymal GBM subtype, a higher glioma grade, and a poorer prognosis [103]. Computational analysis to identify molecular targets in GBMs, looking at differentially expressed genes (DEGs) and differentially expressed miRNAs between GBMs and normal brain tissue and selecting hub genes from miRNA target genes and DEGs, also reveals NRP1 as a gene significantly associated with patient overall survival and disease-free survival [104]. Expression of NRP1, along with several other genes associated with SEMA3 signaling, is significantly associated with four clinicopathological characteristics, namely age, tumor grade, isocitrate dehydrogenase (IDH) mutation, and survival time, and is closely related to an unfavorable patient outcome. A significantly higher rate of patient survival is associated with lower mRNA levels of NRP1 [105]. The presence of the chondroitin sulfate modification in NRP1 is associated with an adverse prognosis in patients with GBM as verified by immunohistochemistry [97].

**Figure 1:**
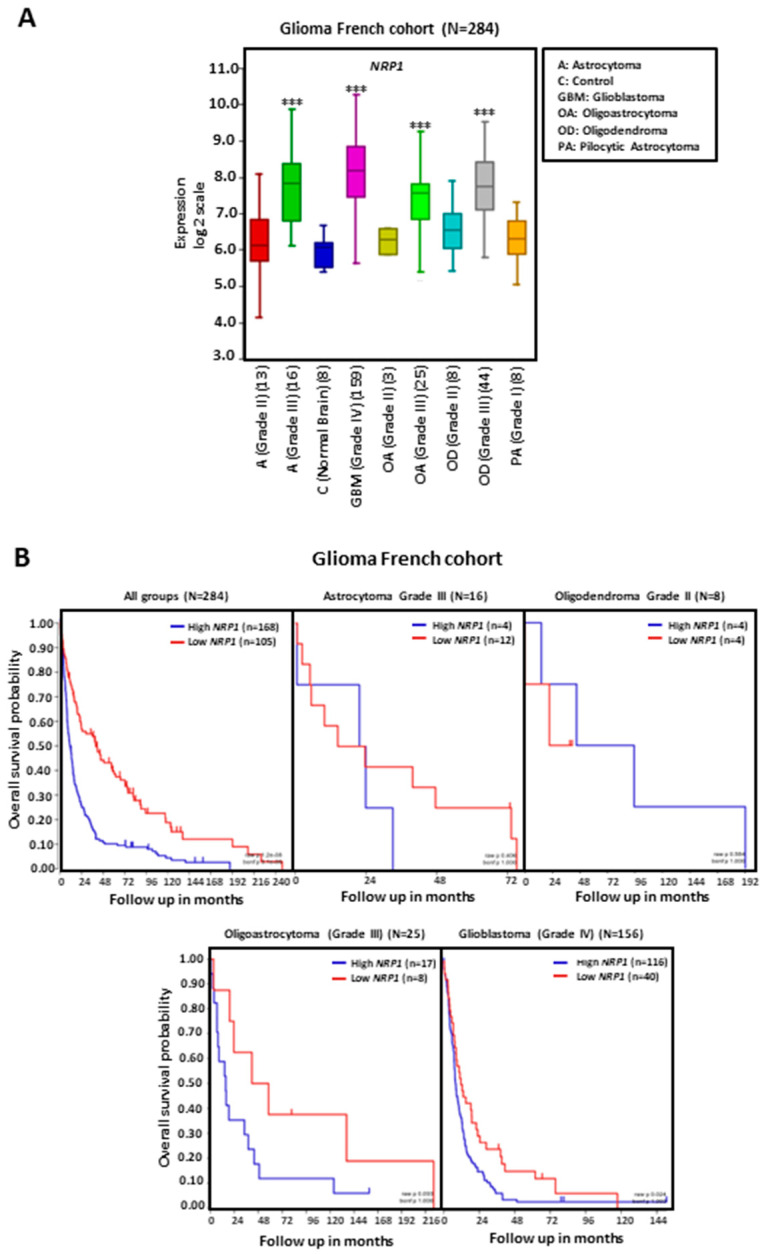
NRP1 gene expression in human glioma. Tumor and normal neural tissue data were obtained from the TCGA French cohort (n = 284) and analyzed with the R2 Genomics Analysis and Visualization Platform (http://r2.amc.nl; accessed on 1 March 2021). Results are presented in boxplot format as log2-transformed signal intensity. (**A**) Bars show data for normal neural tissue (control, C), astrocytoma (**A**) grade II and grade III, GBM, oligoastrocytoma (OA) grade II and grade II, oligodendroma (OD) grade II and grade III, and pilocytic astrocytoma (PA) grade I; *** *p* < 0.001 compared to non-tumoral neural tissue. (**B**) NRP1 expression and overall survival in patients with gliomas. Data for A grade III, OD grade II, OA grade III, and GBM are shown. Patient survival was measured from the day of diagnosis until death or date of last follow-up and calculated using the Kaplan–Meier estimate with median values and long-rank statistics.

**Figure 2:**
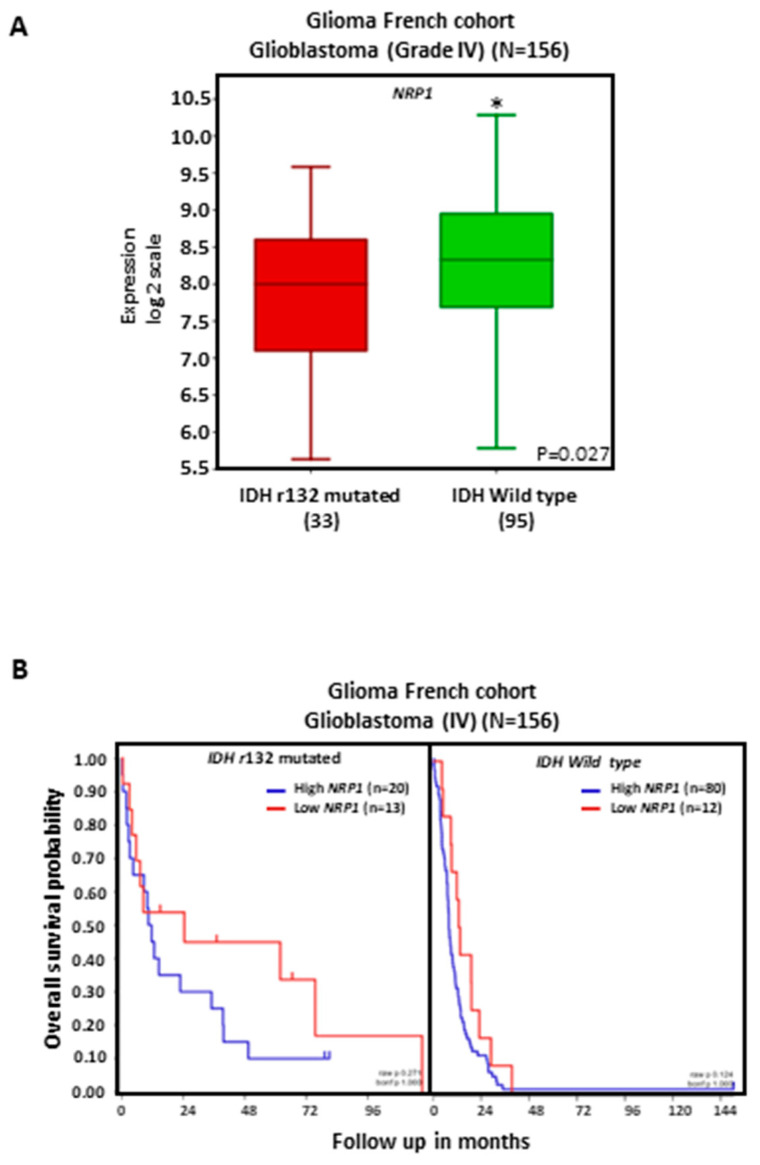
NRP1 expression and overall survival in patients bearing IDH-mutated versus IDH wild-type GBM tumors. GBM tumor data were obtained from the TCGA French cohort (n = 284). (**A**) Results for gene expression are presented in boxplot format as log2-transformed signal intensity; * *p* < 0.05. (**B**) Patient overall survival was measured from the day of diagnosis until death or date of last follow-up and calculated using the Kaplan–Meier estimate.

Analysis of glioma tumors from the TCGA French cohort shows increased levels of NRP1 transcripts in GBMs as well as in other types of higher-grade gliomas, such as grade III astrocytomas, oligoastrocytomas, and oligodendromas, compared to non-tumoral-brain-tissue or lower-grade gliomas (Figure 1A). Increased NRP1 expression is associated with shorter patient overall survival either when glioma types are analyzed together or when only GBM tumors are analyzed (Figure 1B). The NRP1 transcript level is higher in IDH wild-type compared to IDH-mutated GBM tumors (Figure 2A), although this increase does not significantly impact patient survival (Figure 2B).

### 6.2. Lower Grade Gliomas

NRP1 expression is higher in grade III astrocytomas, oligoastrocytomas, and oligodendromas compared to grade I and grade II corresponding glioma tumors (Figure 1A). In astrocytoma cell lines and specimens, the expression pattern of NRP1 is closely associated with more malignant tumors. Mitogens relevant for astrocytoma proliferation, such as EGF and p21-Ras, induce NRP1 expression. Increased NRP1 expression is observed in a transgenic mouse astrocytoma model of astrocytomas [106].

### 6.3. Meningioma

Meningiomas, which account for a little over a third of intracranial cancers, are defined as tumors emerging from the meninges, i.e., the dura mater, arachnoid, and pia mater that envelop the brain and spinal cord. Although most meningioma tumors are benign, they can present grades from grade I to grade III (anaplastic/malignant) associated with aggressiveness. The current WHO classification divides meningiomas into fifteen histologic subtypes across three grades of malignancy that reflect the recurrence rate and prognosis [107,108].

Immunohistochemical analysis of NRP1 expression in a series of 48 cases of meningiomas with different types and histological grades showed NRP1 staining in the vessels within all but two tumors and in the neoplastic cells in eighteen tumors [109]. NRP1 mRNA levels are higher in angiomatous compared to non-angiomatous meningiomas, whereas NRP1 protein levels are lower in angiomatous meningiomas [110,111]. Heterogenous expression of NRP1 was observed in an immunohistochemical analysis performed in tissue microarrays of 232 cranial meningioma specimens from 147 patients, including recurrent tumors. Although associations between the expression of VEGF-A or VEGFR and patient survival were observed, NRP1 did not impact prognosis [112].

### 6.4. Medulloblastoma

A medulloblastoma is the most frequent malignant brain tumor type in children and represents an important cause of cancer-related morbidity and mortality in pediatric patients. Although multimodal therapy consisting of chemotherapy, radiotherapy, and surgery has improved cure rates, about one-third of patients relapse, and survivors show long-term sequalae. Medulloblastomas are classified into four consensus molecular subgroups, namely wing-less-activated (WNT), sonic hedgehog (SHH), Group 3, and Group 4, with patients bearing Group 3 and Group 4 tumors having the worst prognosis. Currently, each subgroup is further classified into twelve subtypes [113,114].

Inhibiting NRP1 function results in antitumor effects in experimental medulloblastomas by influencing cell survival, invasiveness, and stemness [4]. Knockdown of NRP1 reduces the growth of orthotopic human D283 and D341 medulloblastoma xenografts, prevents spinal metastasis, and prolongs survival in mice without affecting cell proliferation by itself. Blocking NRP1 with specific antibodies prevents PlGF-induced activation of protein kinase-mediated signaling pathways in medulloblastoma cells [115]. The peptidomimetic agent MR438, which acts as an NPR1 inhibitor, hinders the self-renewal capacity, invasiveness, and stemness markers in medulloblastoma BTSCs [116] in addition to sensitizing these cells’ radiotherapy in vitro and in vivo [117]. Expression of microRNA miRNA-148a in non-WNT medulloblastoma cell lines reduces NRP1 expression and impairs proliferation, survival, invasiveness, and tumorigenicity. Restoring NRP1 expression rescues miRNA-148a’s effects [118].

An immunohistochemical study showed NRP1 overexpression in five medulloblastoma samples in comparison with non-tumoral pediatric cerebellar tissue. In a set of 32 medulloblastoma tumors, 90% of samples representing different molecular subtypes showed marked expression of NRP1 expression [115]. Another immunohistochemical study using 93 tumor samples showed that patients bearing tumors with moderate or high NRP1 levels had significantly shorter overall survival compared to patients with no detectable or low NRP1 expression [118]. Analysis of a set of 34 tumors with low NRP1 expression and 8 tumors with high NRP1 expression showed reduced survival in patients with high-expressing medulloblastomas [115]. Evaluation of NRP1 transcript levels in a larger medulloblastoma tumor dataset consisting of 763 samples (described by [119]) found widespread expression across samples, with tumors in the SHH subgroup showing higher NRP1 expression in comparison with Group 3 and Group 4 tumors. Unexpectedly, in SHH and Group 3 medulloblastoma tumors, lower NRP1 transcript levels in medulloblastomas are associated with significantly shorter patient overall survival and may indicate poorer prognosis [120].

### 6.5. Ependymoma

Ependymomas are central nervous system (CNS) tumors that arise in the supratentorial region, posterior fossa, or spinal cord and can afflict both children and adults. These tumors are classified on the basis of histological and molecular features, including DNA methylation, age of onset, gender predominance, and the response to therapy. The prognosis for pediatric patients is generally poorer in comparison with adults [121,122]. Analysis via in situ hybridization of the mRNA for NRP1 found its expression in grade II and grade III ependymoma samples. NRP1 expression was associated with tumor cells rather than tumor vessels and increased with grade in the tumor vessels [123].

## 7. Conclusions

The evidence reviewed above consistently indicates that, in many instances, increased expression of NRP1 predicts poorer patient outcomes as indicated by a shorter overall survival. In brain tumors, this is observed in GBMs ([80,88,91], present article) and medulloblastomas [115,118], although specifically in SHH and Group 3 tumors, shorter patient survival may be related to lower NRP1 transcript levels [120]. In order to be clinically useful, predictive biomarkers need to be validated through a strictly regulated multi-step process involving specific trial designs and statistical approaches. The current knowledge on NRP1 expression in cancer provides an early step indicating that the potential of NRP1 as a biomarker should be further investigated in large tumor cohorts with special attention to the possible differences among distinct molecular subgroups within each tumor type.

Current evidence also indicates that inhibiting NRP1 activity through any of the different possible approaches, from small molecule-mediated inhibition to RNA interference knockdown, can display antitumor effects in several cancer types and across a range of experimental models and conditions. The fact that NRP1 is a transmembrane glycoprotein makes it a potentially more “druggable” target compared to, for example, transcription factors or other components of the intracellular machinery. Although most of the work performed on NRP1 inhibition in cancer to date remains at the preclinical stage, clinical research initiatives have emerged. Thus, the findings of a stimulating role of the PlGF/NRP1 pathway in pediatric cancers have led to an open-label Phase I clinical trial aimed to evaluate TB-403, a monoclonal antibody against PlGF, in pediatric patients with relapsed or refractory MBs, neuroblastomas, Ewing sarcoma, or alveolar rhabdomyosarcoma [124]. The ongoing progress in experimental therapy will likely allow the development of a pipeline of NRP1 inhibitors feasible for clinical testing in patients with brain cancers. The example of NRP1 highlights the potential of regulators of nervous system development as biomarkers and molecular targets in brain tumors.
